# Vertical transmission: evidence of COVID-19 in a twin pregnancy

**DOI:** 10.5935/1518-0557.20210058

**Published:** 2022

**Authors:** Héctor Salvador Godoy Morales, Daniel Vieyra Cortés, Hilda Sanchez Hernández, Miguel Loyo Guiot, Griselda Claribel Reyes Torres, Francisco Miguel Rojas Camacho, Gabriela Ayala Montoya, Berenice Flores Maldonado, José Guzmán Bárcenas, Germán Gabriel Palacios López

**Affiliations:** 1 Head of Assisted Research and Treatment in Human Reproduction (ART) Reproductive Medicine Unit at Ángeles del Pedregal Hospital, Mexico City, Mexico; 2 Specialist in Human Reproductive Medicine at Ángeles del Pedregal Hospital, Mexico City, Mexico; 3 Resident of Human Reproductive Medicine at Ángeles del Pedregal Hospital, Mexico City, Mexico; 4 Specialist in Neonatology at Ángeles del Pedregal Hospital, Mexico City, Mexico

**Keywords:** vertical transmission, placenta, COVID-19, neonate, pre-term

## Abstract

This article reports the case of a 28-year-old female 31.6 weeks pregnant with twins diagnosed with SARS-CoV-2 infection, who delivered a boy and a girl. The newborns underwent RT-PCR testing for SARS-CoV-2; the male tested negative and the female newborn tested positive, in that the female placenta was SARS-CoV-2 positive and the male placenta negative. Clinical and laboratory findings evincing vertical transmission of SARS-CoV-2 were identified. Strict, multidisciplinary prenatal care is recommended for this group of patients. This case report alone does not provide statistical evidence of vertical transmission, but it is an account of a relevant matter.

## INTRODUCTION

Despite discussions about the risk of vertical transmission of COVID-19, there is no globally accepted screening protocol for the diagnosis and management of mothers and fetuses beyond reverse transcription polymerase chain reaction (RT-PCR) testing (Karimi-Zarchi *et al*., 2020).

Over the course of this pandemic, we have noticed that pregnant women and their fetuses should be categorized as a high-risk infection group considering that most with preexisting comorbidities might present mild symptoms (Mehan *et al*., 2020; Smith *et al*., 2020; Dashraath *et al*., 2020). They may develop severe clinical involvement requiring referral to an intensive care unit, although deaths have been rarely reported (Dashraath *et al*., 2020; Chen *et al*., 2020; Elshafeey *et al*., 2020). The immune system during pregnancy has more T-helper 2 (Th2) anti-inflammatory activity than T-helper 1 (Th1) microbicidal lymphocyte activity, which leads to greater susceptibility to viral respiratory infections. Patients with COVID-19 have increased Th1 and Th2 response two weeks after the onset of infection, and present with extended lung damage and risk of death when elevated levels of Interleukin-6 (IL-6) are detected (Ruan *et al*., 2020).

Angiotensin-converting enzyme 2 (ACE2) functions as a severe acute respiratory syndrome coronavirus 2 (SARS-CoV-2) receptor and allows the virus to infect respiratory epithelial cells (Levy *et al*., 2008). Although some authors did not find SARS-CoV-2 in placenta, amniotic fluid, breast milk or cord blood (Chen *et al*., 2020; Elshafeey *et al*., 2020), others did and established vertical transmission as a possibility (Penfield *et al*., 2020).

## CASE REPORT

A 28-year-old non-obese female came to our Center with male factor (vasectomy) and a history of two years of primary infertility and underwent in vitro fertilization (IVF) and preimplantation genetic testing (PGT). Two chromosomally normal embryos, one female and one male, were transferred after endometrial preparation. Two gestational sacs were identified 20 days after embryo transfer and vitality was confirmed in both embryos at 6.3 weeks. The patient had been isolated at home since week 18, when social distancing measures were implemented in Mexico City due to the COVID-19 pandemic. Prenatal control was normal until week 28, when fetal growth restriction was observed. She was prescribed betamethasone for lung maturation.

At week 30, she presented a flu-like infection with rhinorrhea, mild nasal congestion, otalgia, odynophagia, and productive cough without fever or respiratory distress. Her oxygen saturation ranged from 95% to 98% during symptom presentation. At 31.6 weeks, Ultrasound Doppler Flowmetry (UDF) images indicated the female suffered from fetal growth restriction and blood flow redistribution; the male twin had a normal UDF. The twins had their heart rates monitored. A diagnostic protocol for COVID-19 was initiated with complete viral profile, nasopharyngeal swab RT-PCR testing, and chest computed tomography (CT) scans due to persistent respiratory symptoms (Table 1). The RT-PCR tests of both parents were positive for COVID-19.

**Table 1.** Clinical case findings.

Findings	Mother	Male Twin	Female Twin
Symptoms	Rhinorrhea, productive cough without fever	-	Fetal tachycardia
RT-PCR: 7/4/2020	Positive	Negative	Positive
RT-PCR: 7/13/2020	Negative (breast milk)	Positive	Positive
Laboratory	Lymphopenia		
Blood Type		O+	A+
CT findings	Polished glass bilateral images basal pneumonitis consistent with COVID-19	***	***
IgG/IgM	Positive /Negative	Both negative	Both negative
Morbidity	***	Pre-term birth	Myocarditis
Placental histopathology findings	***	Intervillous fibrin hypertrophic decidual vasculopathy.	1 cm villous infarction hypertrophic decidual vasculopathy intervillous fibrin
RT-PCR: placenta	***	Negative	Positive

The management protocol for COVID-19 was started with isolation and a requirement for healthcare workers assigned to the patient to wear Category III Personal Protective Equipment (Category III PPE). From the start of respiratory symptoms - one week before hospitalization − until delivery, the patient remained without evidence of respiratory distress or hemodynamic involvement. The patient was prescribed azithromycin, dexamethasone, and hydroxychloroquine (as indicated by the Department of Infectious Diseases, based on the protocol in effect at the hospital at the time). Fetal heart rate monitoring (FHRM) found tachycardia of 180 bpm and 150 bpm with decreased variability in the female and male fetuses. The female fetus had growth restriction, blood flow redistribution, and tachycardia, and a Cesarean section was performed at week 32 under epidural block. Medical personnel wore Category III PPE and only healthcare workers deemed essential were given access to the operating room. With both amniotic sacs intact, first the male cephalic twin and then the female pelvic twin were delivered, aspirated, and handed separately to two neonatologists. The twins were kept in isolation in a COVID-19 neonate room and did not have skin-to-skin contact with the mother. The male twin weighed 1780 grams and had an Apgar score of 9 at one minute and 9 at 5 minutes. The female twin weighed 1020 grams and had an Apgar of 9 at 1 minute and 9 at 5 minutes. Both twins needed endotracheal intubation for pulmonary surfactant administration. After 12 hours, they were extubated and received oxygen for the following 24 hrs. The female twin had persistent tachycardia and myocarditis, possibly secondary to COVID-19 infection.

Since hospital admission, the patient wore an N95 mask, and even during surgery she did not hold the neonates; a different team of neonatologists handled each twin. The patient improved gradually and without respiratory distress. Enoxaparin and ivermectin were administered in the immediate postoperative period. She was discharged 36 hours after delivery with mild symptoms and continued in isolation. The mother was IgG-positive and IgM-negative five days after delivery. Samples of nasal, pharyngeal, and bronchial aspirates taken from the female twin tested positive for COVID-19 in RT-PCR testing, while male twin was negative. Biological samples were collected immediately after birth with an aseptic technique (within less than an hour). The RT-PCR tests of the male twin and his placenta tested positive for COVID-19; breast milk tested negative seven days after birth.

## DISCUSSION

To our knowledge, there is no strong evidence to confirm the presence of vertical transmission for SARS-CoV-2 (Elshafeey *et al*., 2020; Dashraath *et al*., 2020). However, we have noticed that vertical transmission is possible since ACE 2 receptors have been found in the uterus and placenta (Levy *et al*., 2008). The latter might explain why the placenta of the infected neonate was positive and the placenta of the other newborn was negative. Penfield *et al*. (2020) support our theory, since they found the virus in amniotic fluid and placenta.

**Figure 1:**
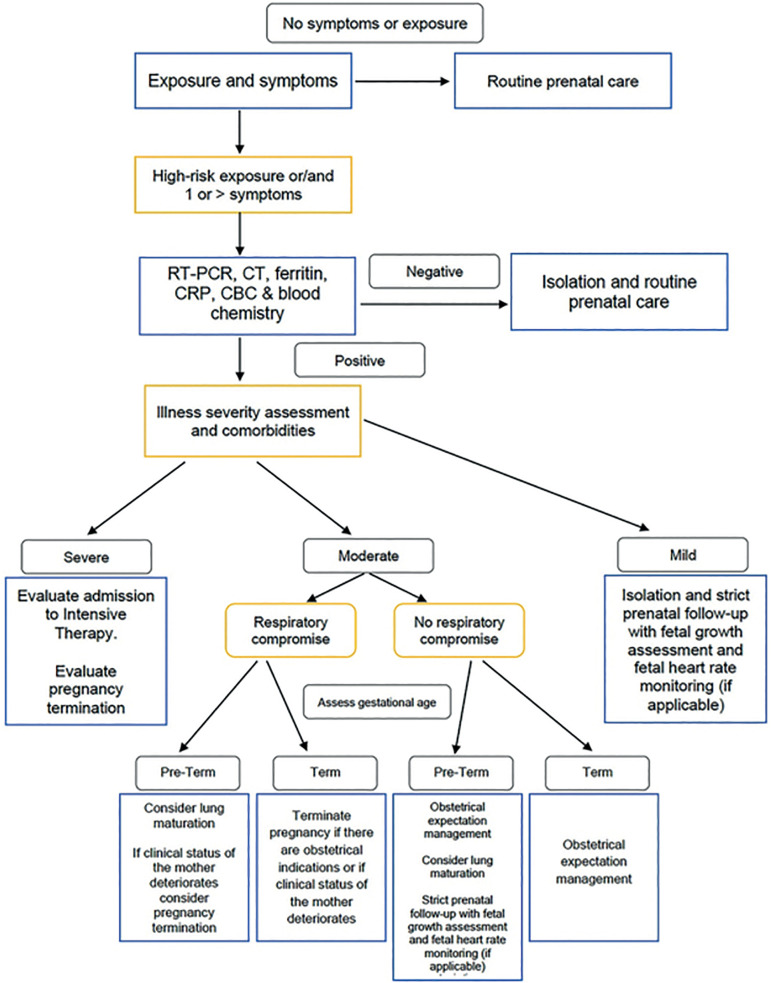
Algorithm for SARS-CoV-2 Detection in Pregnancy.

The suggestion of an ideal diagnostic protocol for SARS-CoV-2 initiated discussions around adequate approaches considering the scope and timing to treat neonates and mothers with COVID-19 (He *et al*., 2020). Since SARS-CoV-2 infection has unfortunately become the leading cause of maternal death in our nation (Gobierno de México, 2020), we propose a protocol for the early diagnosis of pregnant women to thus reduce perinatal complications (Figure 1).

**Figure 2:**
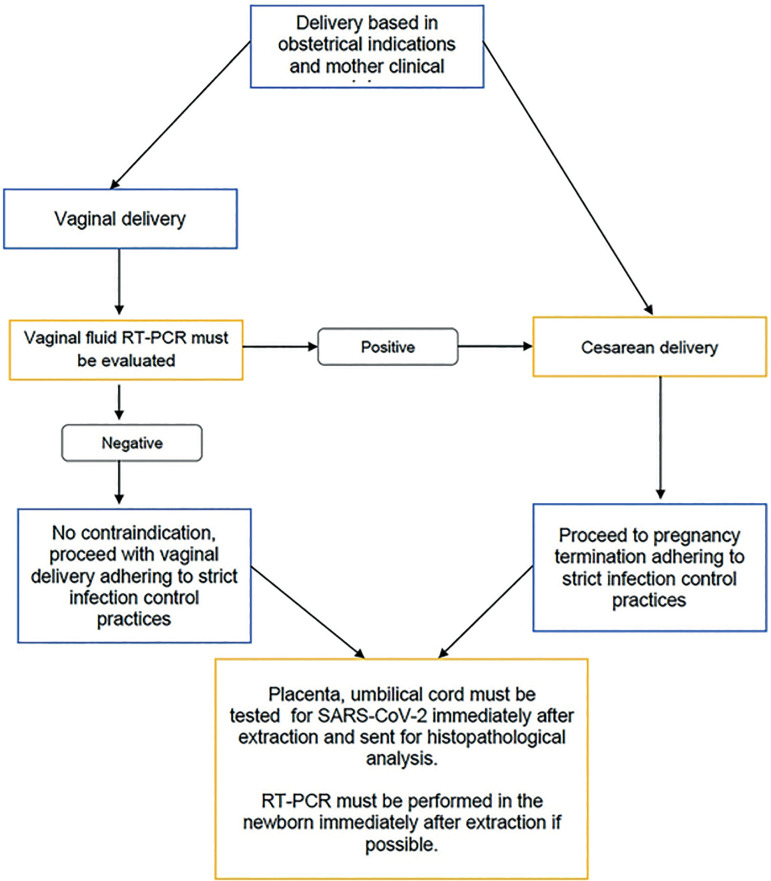
Algorithm for Detection of Possible Vertical Transmission.

Likewise, we would like to stress the importance of timing in SARS-CoV-2 RT-PCR testing for neonates, and that newborns be tested immediately after birth. Histopathology examination and immunohistochemical analysis of the placenta and bronchial lavage are also possible sources of analysis for SARS-CoV 2 infection (Figure 2), since fetal growth restriction has been associated with maternal SARS-CoV-2 infection in the third trimester of pregnancy; hence, the importance of an adequate protocol for the diagnosis and early detection of fetal and maternal complications.

## CONCLUSIONS

Strict, multidisciplinary prenatal care is recommended for this group of patients, including growth monitoring (to rule out FGR), structural ultrasound examination, and rigorous pediatric care to prevent future morbidity. This case report alone does not provide statistical evidence of vertical transmission, but it is an account of a relevant matter.
